# Scanning electron microscopy and morphometric analysis of the hair in dromedaries with SEM-EDX in relation to age

**DOI:** 10.1186/s40850-024-00204-0

**Published:** 2024-07-15

**Authors:** Mohamed A.M. Alsafy, Samir A.A. El-Gendy, Amira Derbalah, Ahmed M. Rashwan, Seham S. Haddad

**Affiliations:** 1 Department of Anatomy and Embryology, Faculty of Veterinary Medicine, Alexandria University, P.O. 21944, Abis 10th, Alexandria, Egypt; 2 Histology and Cytology Department, Faculty of Veterinary Medicine, Alexandria University, P.O. 21944, Abis 10th, Alexandria, Egypt; 3 Department of Anatomy and Embryology, Faculty of Veterinary Medicine, Damanhour University, Damanhour, 22511 Egypt; 4 Laboratory of Life Science Frontiers, Center for iPS Cell Research and Application (CiRA), Kyoto University, 53 Kawahara-cho, Shogoin, Sakyo-ku, Kyoto, 606-8507 Japan; 5 Department of Anatomy and Embryology, Faculty of Veterinary Medicine, University of Sadat City, Sadat City, 32897 Egypt

**Keywords:** Maghrebi camel (*camelus dromedarius*), Hair, Light microscopy, SEM, EDX

## Abstract

**Background:**

Hair characterization is critical for determining animal individuality throughout life. This study aimed to assess the morphological features of dromedary camel hair in relation to age.

**Materials and methods:**

Hair samples were obtained from the camel humps of 30 dromedary camels separated into three groups: G1 (n:10) aged one-year, G2 (n:10) aged 3–5 years, and G3 (n:10) at the age of 8–10 years. The hair was examined using light microscopy, SEM, and SEM-EDX.

**Results:**

The Maghrebi camel had varied medulla patterns and structures across the ages. In the G1 group 75% had continuous medulla patterns and amorphous medulla structures, compared to 70% in G2, and 90% in G3. The medulla index increased with age, rising from 0.3 to 0.77%. The shaft width grew in size from G1 to G2, then fell to approximately one-third of the G2 size at G3. The cortex and cuticle widths were also determined by age, and they increased in the G1 compared to G3 camels. The shape of the cuticle scales in G1 camels was wavy, like mountain tops with irregular edges, within G2 camels the scales were particularly long, oval-shaped scales with smooth, wavy borders. The scales of the older G3 camels were quite long and rectangular. SEM-EDX spectra recognized carbon, oxygen, nitrogen, sulfur, calcium, aluminum, silicon, and potassium at the medulla and cortex. Sulfur levels were highest in the G2 samples but lowest in the G1 samples.

**Conclusion:**

The dromedary camel’s hair structure and mineral content, particularly carbon and nitrogen, differed as camels aged.

## Introduction

The camel is a multipurpose animal used for meat, milk, hair, transportation, agricultural work, racing, and tourism [1–4]. Camels belong to the order Artiodactyla, sub-order Tylopod, and family *Camiladae*. There were two genera within the family genus *Camelus; Camelus dromedarius*, commonly called the dromedary camel (one hump), and *Camelus bactrianus*, the Bactrian camel (which has two humps) [5, 6]. Camels in Egypt belong to the *Camelus dromedarius* family, also known as Arabian camels [6–8]. Egypt has five camel populations: Maghrebi, Somali, Sudani, Baladi (Fallahi), and Mowallad. Three populations (Baladi, Maghrebi, and Sudani) were discovered to have a high degree of genetic purity with a low degree of mixing, so they are considered pure populations [9, 10]. Mowallad is a hybrid of Maghrebi and Falahi [11]. These camel populations are used for a variety of purposes. The Sudani are commonly used for riding and racing, the Fallahi, also called Baladi are often used for transportation and agricultural operations, whilst Maghrebi are dual-purpose as they are often used for meat and milk [2, 11, 12].

In criminal investigations, hair inspection is used as typical physical evidence; it is essential to ascertain if the hair is of human or animal origin and to identify the animal species [13, 14]. Hair is stable under adverse natural conditions, making it useful in forensic investigations and resistant to postmortem changes such as chemical and environmental degradation [15]. The use of scanning electron microscopy (SEM) as a rapid method for the identification of variation in the morphological aspects of hair in animals is useful to understand scale features, patterns, and diameters as a key component of differentiation [16, 17]. Additionally, recent work [18] has helped uncover three-dimensional visualization of the hair scale pattern and the medulla and cortex.

Camel hair, also known as wool and cashmere, are two animal fibers that have been extensively explored and manufacturing techniques have been well developed [19, 20]. Camel wool is a preferred material for clothing and other items because of its flexibility, toughness, high quality, wear resistance, excellent insulation, and warmth compared to sheep wool [21]. Camel hair fibers are categorized as specialty hair fibers because they are rare animal fibers with distinctive properties such as luster, softness, warmth, and natural color [22].

Hair comprises of an inner medulla, a middle cortex, and an outer cuticle in all species. With the exception of dogs, all animals have a noncellular cortex and a cellular medulla formed of cornified cells [23]. Dromedary camels have thin, non-medullated, or medullated hair fibers and also thick, interrupted, or continuous medulla hair fibers [24]. Hairs also contain scales, which are unassuming sutures used to identify animal hairs. Fiber scales play an important role in reflecting sunlight, the type of glint of the hair, and configure industrial characteristics such as smoothness, luster, felting, and friction [25, 26]. To study these aspects, and others, hair has been studied using modern methods such as ion mobility spectrometry, Energy Dispersive X-ray spectroscopy (EDX), which has showed significant differences in hair concentrations of trace elements, and scanning electron microscopy has also been used to provide precise morphological examination of the structures [16, 27, 28].

The camel is a symbol of culture and civilization in many parts of the world. The camel’s ability to live in harsh conditions with limited food supplies and a hot environment has inspired research into their hair to investigate the detailed architecture of dromedary camel hair. We investigated hair variations by animal age using advanced microscopic tools. Therefore, our study aimed to determine the morphological characteristics of Maghrebi camels of three different ages living in Egyptian conditions, which would be useful for forensic purposes and industrial applications.

## Materials and methods

### Samples

All methods followed relevant guidelines and regulations with ethical permission from the Alexandria University Research Ethics Review Committee of the Faculty of Veterinary Medicine, Alexandria University (*Approval No: Au/13/12/12/2023/059*).

**Fig. 1:**
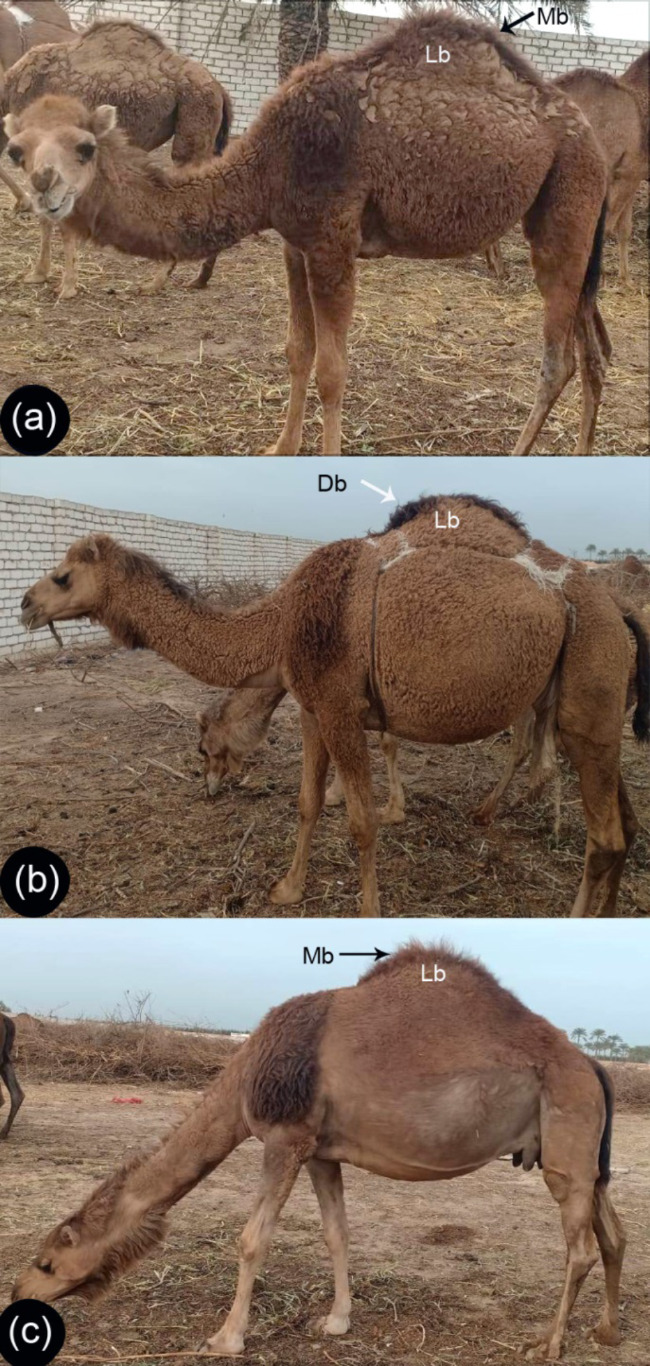
A photograph of a Maghrebi camel: Examples of (**A**) G1, (**B**) G2, and (**C**) G3 camels. The hair at the center of the hump was medium brown (Mb) in G1 and G3 camels and dark brown in G2, and the rest of the hump had light brown (Lb) hair in all three groups

Hair samples from the hump region only were collected from 30 Maghrebi one-humped camels (*Camelus dromedarius*) from the Menoufia Governorate, Egypt. The three age groups were collected and classified into three groups, young (G1 one year), pre-puberty (G2 3–5 years), and puberty (G3 8–10 years) with 10 animals per group (Fig. 1). The hairs were collected from the camel humps from both sexes (15 male and 15 female). Camels were excluded from sampling if they had dirty, corrupt, and incomplete hair. The hair samples were cleaned with distilled water and placed in 70% ethanol for 10 min.

### Light microscopic (LM) examination and measurements

The samples were placed on a clean microscope slide. Then, they were mounted in Canada balsam (MilliporeSigma™ USA) and examined under a light microscope at various magnifications [29, 30]. The diameter of the hair shaft, medulla, and cortex were measured micrometrically using an Olympus CX31 camera-mounted microscope and Image Pro Express Ver-2.0 software, and data was analyzed using standard statistical methods [16].

### Scanning electron microscopic (SEM) examination and measurements

Hair samples were taken from the middle of the hair and fixed in a buffered solution (2% formaldehyde and 1.25% glutaraldehyde in a 0.1 M sodium cacodylate buffer, pH 7.2) at 4^o^C. After being fixed, the samples were washed in 0.1 M sodium cacodylate containing 5% sucrose before being dehydrated in increasing ethanol concentrations (15 min in 50, 70, 80, 90, 95, and 100% ethanol). They were Critical Point Drying (CPD) treated, colloidal carbon-attached to stubs, and gold-palladium-coated in a sputtering device (JFC-1100E), with a gold coat thickness of about 5–10 nm [31, 32]. Specimens were examined and photographed in a JEOL JSM-IT200 scanning electron microscope at 15 kV at the electron microscope unit in the Faculty of Science, Alexandria University [33].

The obtained SEM images were investigated via ImageJ (Wayne Rasband and contributors, National Institutes of Health, USA) [34, 35]. The measurements taken were the different diameters of the hair shaft, medulla, and cortex, and the means ± standard errors were calculated for each parameter for each group and P value were calculated. The data were analyzed using one-way analysis of variance (ANOVA) using SPSS version 21. Duncan’s multiple range test was used to test the significance of the differences between the mean values. Statistical significance was set at *P* < 0.05.

### SEM-energy dispersive X-Ray analysis (SEM-EDX)

The samples from the hair cuticles and cross-sections of hairs from the three age groups of camels were analyzed and photographed using a JEOL JSM-IT200 scanning electron microscope at 20 kV. The quantification method used was ZAF analysis. The sample distance from the detector was 10 mm, the real-time was 30.97 s, and the dead time was 3.00%. The EDX spectrometer was created at the Science Faculty, Alexandria University, Egypt [36, 37].

## Results

Overall, the hair at the center of the hump was medium brown in color in the G1 and G3 camels, while they were dark brown in the G2 animals. The rest of the hump had light brown hair coloration in all three groups (Fig. 1).

### LM examination

**Fig. 2:**
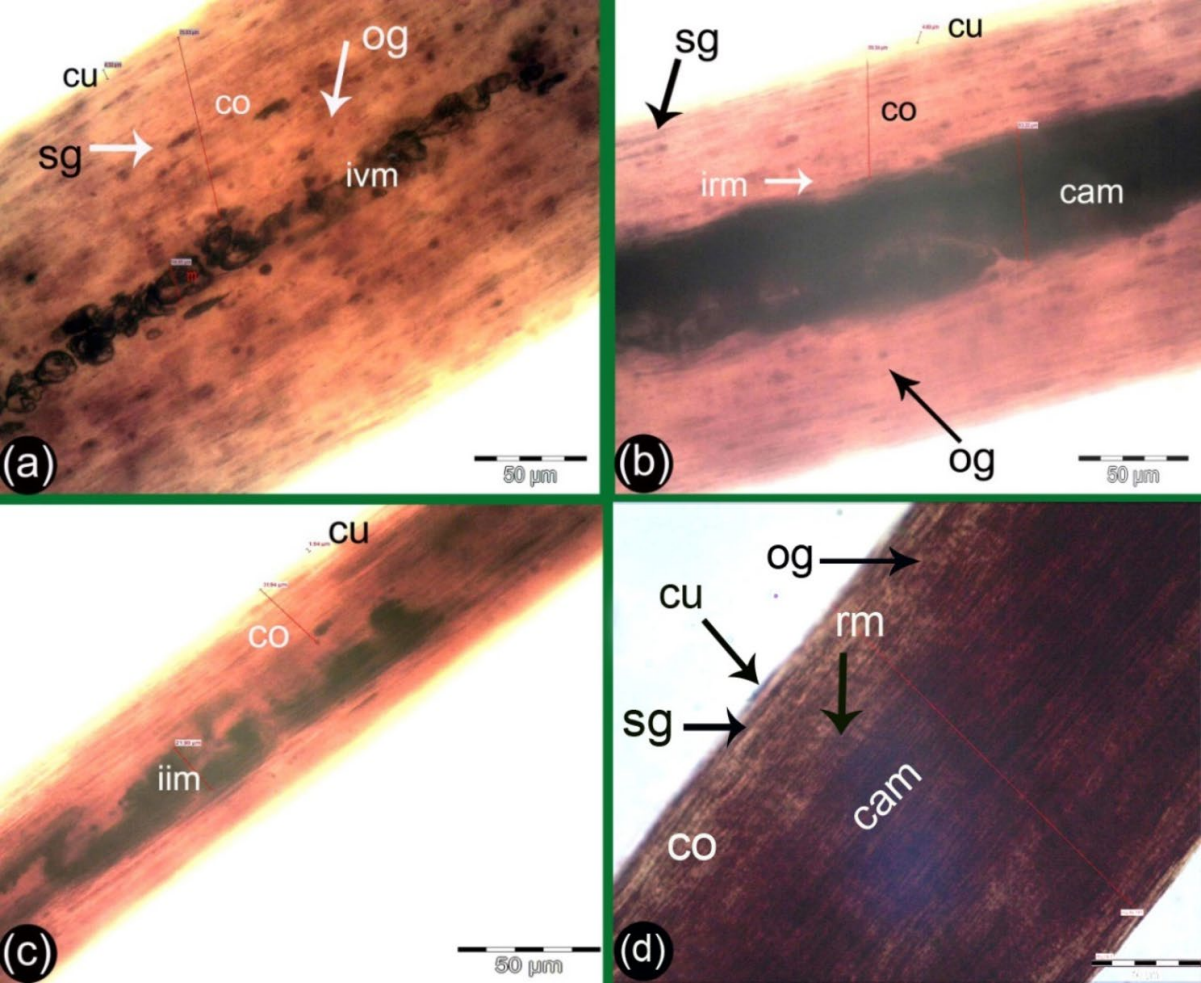
Photographs showing the basic structure of the hair shaft and the measurements of the medulla of the G1 of camels. The hair in panel d was from the center of the hump and had a moderate brown color, while the hair in panels a, b, and c were from the rest of the hump and had a light brown color. Hair cuticle (cu), cortex (co) with small oval pigment (ov) or streak-like pigment (sg) and different shapes of medulla pattern, panel (**a**) showed very thin medulla with interrupted pattern, vacuolated structure, and irregular margins (vim), panels (**b** and **d**) the medulla showed continuous pattern, amorphous structure (cam) and irregular margins (irm), and panel (**c**) showed interrupted intruding pattern and amorphous structure (iim)

**Fig. 3:**
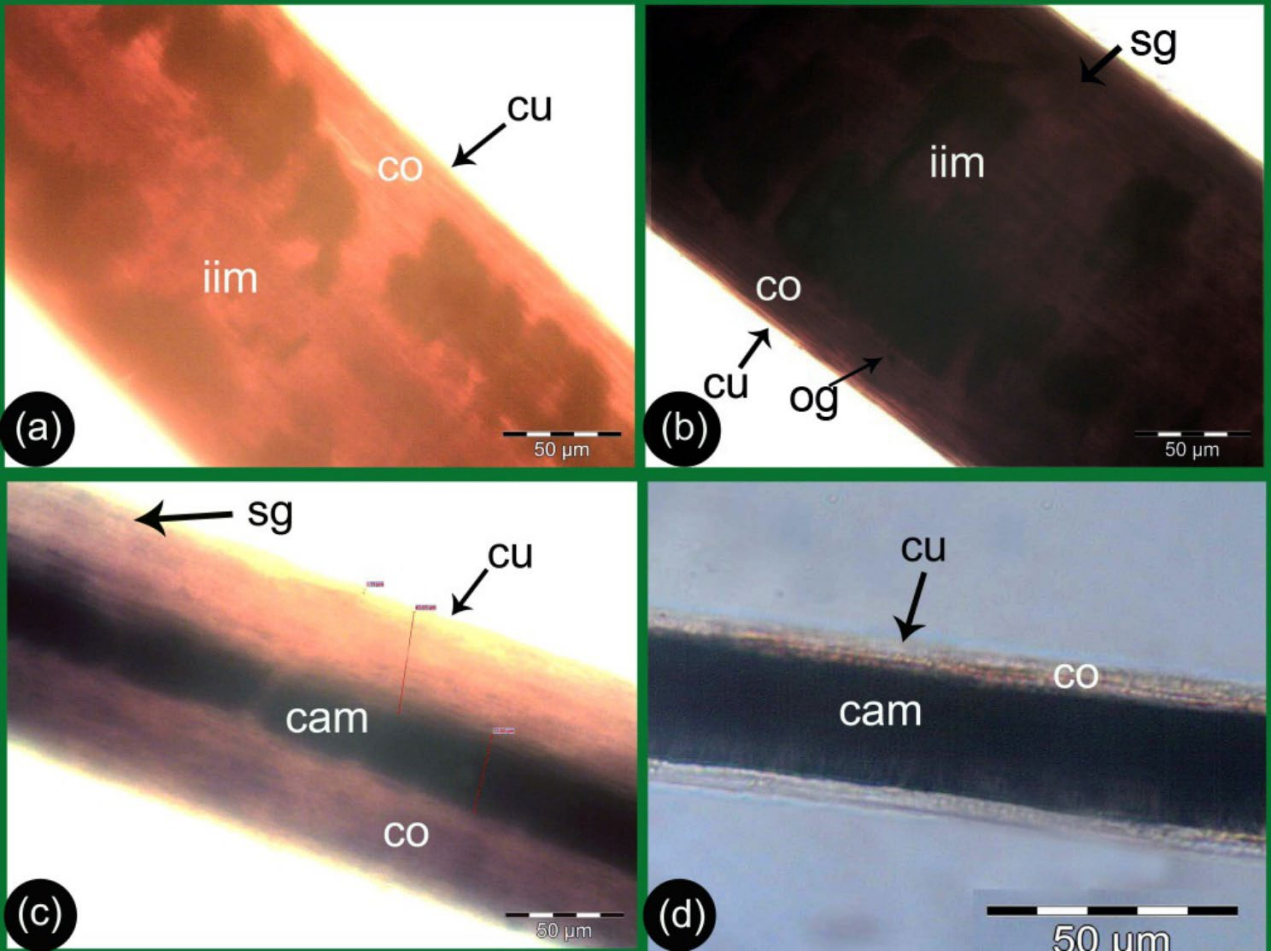
Photographs showing the basic structure of the hair shaft and measurement of the medulla of the G2 camel. The hair in panel d was from the center of the hump and had a dark brown color, while the hair in panels a, b, and c were from the rest of the hump and had a light brown color. Hair cuticle (cu), cortex (co), small oval pigment (ov), streaks like pigment (sg), and different shapes of medulla pattern, panels (**a** and **b**) showed interrupted intruding medulla pattern with amorphous structure (iim), panels (**c** and **d**) of the medulla showed continuous pattern and amorphous structure with irregular margin (cam) in panel (**c**) and regular margins were present in panel (**d**)

**Fig. 4:**
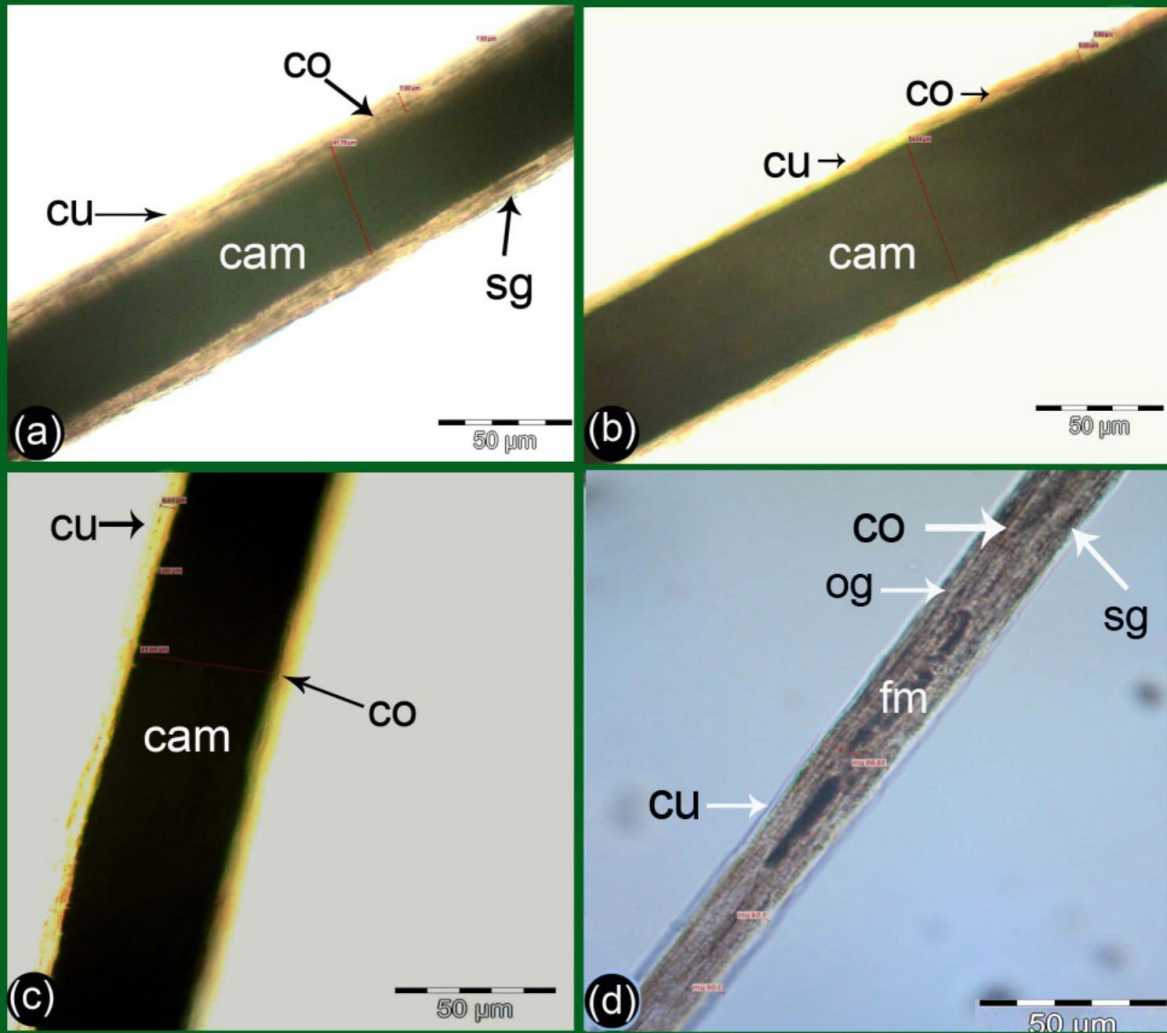
Photographs showing the basic structure of the hair shaft and measurement of the medulla of the G3 camel. The hair in panel (**d**) was from the center of the hump and had moderate pigmentation and moderate brown color, while the hair in panels (**a**, **b**, and **c**) were from the rest of the hump and had low pigmentation and light brown color. Hair cuticle (cu), cortex (co) with streaks like pigment (sg), oval shape pigment (og). In panels (**a**, **b**, and **c**), the medulla was a wide continuous pattern and amorphous structure with regular margins (cam). In panel (**d**) the medulla pattern was fragmented and had an amorphous structure with irregular margins (fm)

Light microscopy revealed that the medulla pattern appeared to be either continuous (Fig. 2a), interrupted (Fig. 3a, b), or fragmented (Fig. 4d) in the different age stages. The medulla structures appeared vacuolated (Fig. 2a), intruding, and amorphous (Fig. 2c). However, the medulla margin appeared to be either regular or irregular (Fig. 2b, d). These properties are all detailed below for each group.

The hairs from the G1 camels were light in color. In 75% of the G1 camels, the medulla had a thin continuous pattern and an amorphous structure with regular and irregular margins (Fig. 2b, d); however, in the remaining 25% the medulla had a thin interrupted pattern (Fig. 2c) and vacuolated structures with regular and irregular margins (Fig. 2a). The hair of the central part of hump had moderate pigmentation in this younger group. It appeared moderate brown in color (Fig. 2d, ) whilst hairs from the peripheral regions of the hump had light brown coloration with lower pigmentation observed (Fig. 2a, b).

The cortex within the hair from the G2 camels appeared dark brown with small oval pigments and large granules that seemed to be streak-like from hair from the center of the hump (Fig. 3b). In contrast hair from the peripheral of the hump appeared light in color brown (Fig. 3a), and 70% of these camels, had medullas with a continuous pattern and amorphous structure with irregular and regular margins (Fig. 3c, d), meanwhile, the remaining the medulla in the remaining 30% of camels had an interrupted medulla pattern with interrupted intruding structures (Fig. 3a, b).

The cortex from the G3 camels from the central part of hump had moderate pigmentation and appeared to have moderate brown coloration of the hair (Fig. 4d), whilst the hair from rest of hump was light brown with lower pigmentation (Fig. 4a, b, c). In 90% of camels, the medulla had a wide continuous pattern and an amorphous structure with regular margins on (Fig. 4a, b. c), on the other hand, the other 10% exhibited a thin fragmented medulla pattern with an amorphous structure (Fig. 4d).

### LM measurements

**Chart 1:**
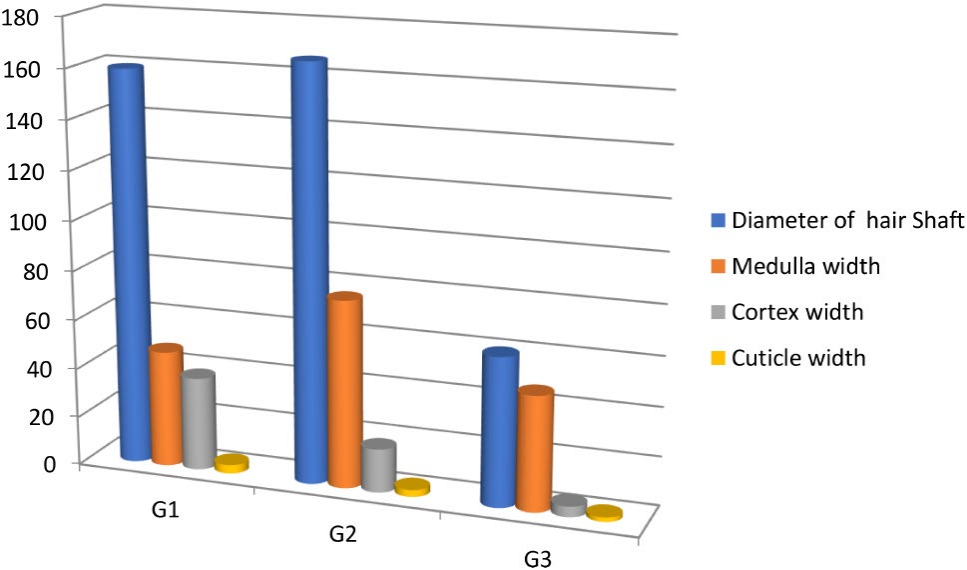
Change in camel hair diameter, medulla, cortex, and cuticle width at three different ages using light microscope analysis. Whereas the diameter of the shaft and medulla width at G2 were at their highest values, and the diameter of the cortex and cuticle had the highest values in G1

The shaft width increased from G1 to G2, from 159.4 ± 7.59 μm to 166.48 ± 7.31 μm, and a significant decrease (p-value = 0.0499) in shaft width at G3 to about one-third of the previous ages, to just 60.46 ± 2.39 μm. With increasing age, the width of the cortex and cuticles decreased (p-value = 0.0321 and 0.0398 respectively) and was mostly observed in the G1 camels (Table 1 and Chart 1).

**Table 1:** Camel hair width of the shaft, medulla cortex, and medulla index at the three different ages by light microscope (mean ± standard error and p-value)

Measurements	G1	G2	G3	*p*-value
Diameter of the hair shaft (width) (µm)	159.25 ± 7.59^a^	166.50 ± 7.31^a^	60.46 ± 2.39^b^	0.0499
Medulla width (µm)	47.44 ± 3.59^b^	76.02 ± 7.77^a^	46.99 ± 2.53^b^	0.0506
Medulla index %	0.298 ± 0.01^c^	0. 456 ± 0.02^b^	0. 772 ± 0.05^a^	0.0491
Cortex width (µm)	38.11 ± 2.10^a^	17.84 ± 2.15^b^	4.31 ± 0.30^c^	0.0321
Cuticle width (µm)	3.51 ± 0.17^a^	2.80 ± 0.48^b^	1.80 ± 0.52^c^	0.0398

Means within the same row, different superscripts are significantly different at *P* < 0.05

The medulla index increased significantly (p-value = 0.0491) across all three ages in the three groups of camels, varying from 0.298 ± 0.01 (G1) to 0.456 ± 0.02 (G2) to 4.31 ± 0.3% (G3), with G3 showing the greatest increase (Table 1 and Chart 1).

### SEM examination

**Chart 2:**
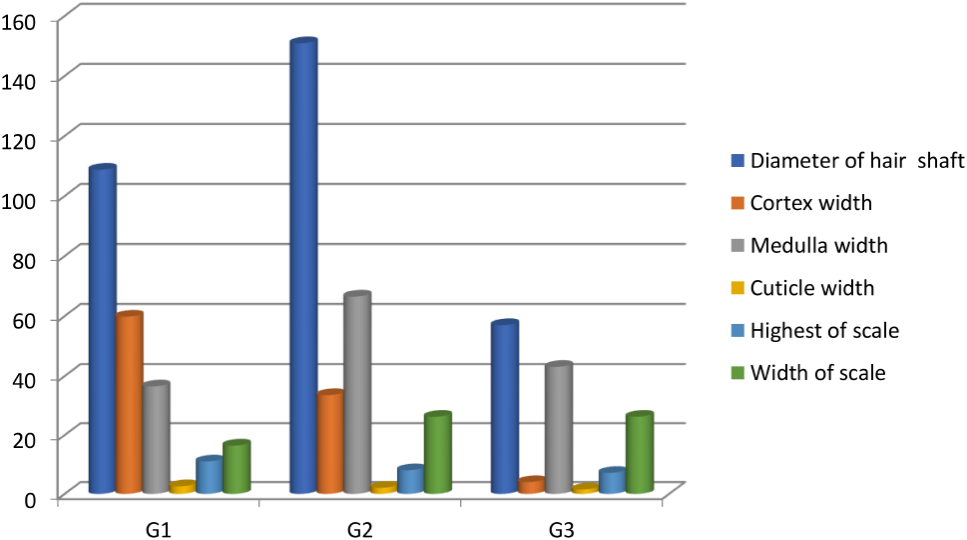
Changes of camel hair width in the shaft, medulla, cortex, cuticle, and scale measurements at the three different ages via SEM analysis. Whereas the diameter of the shaft and medulla width at G2 were the highest values, the diameter of the cortex and cuticle were highest in G1, while the width of the scale was the highest value at G3

**Fig. 5:**
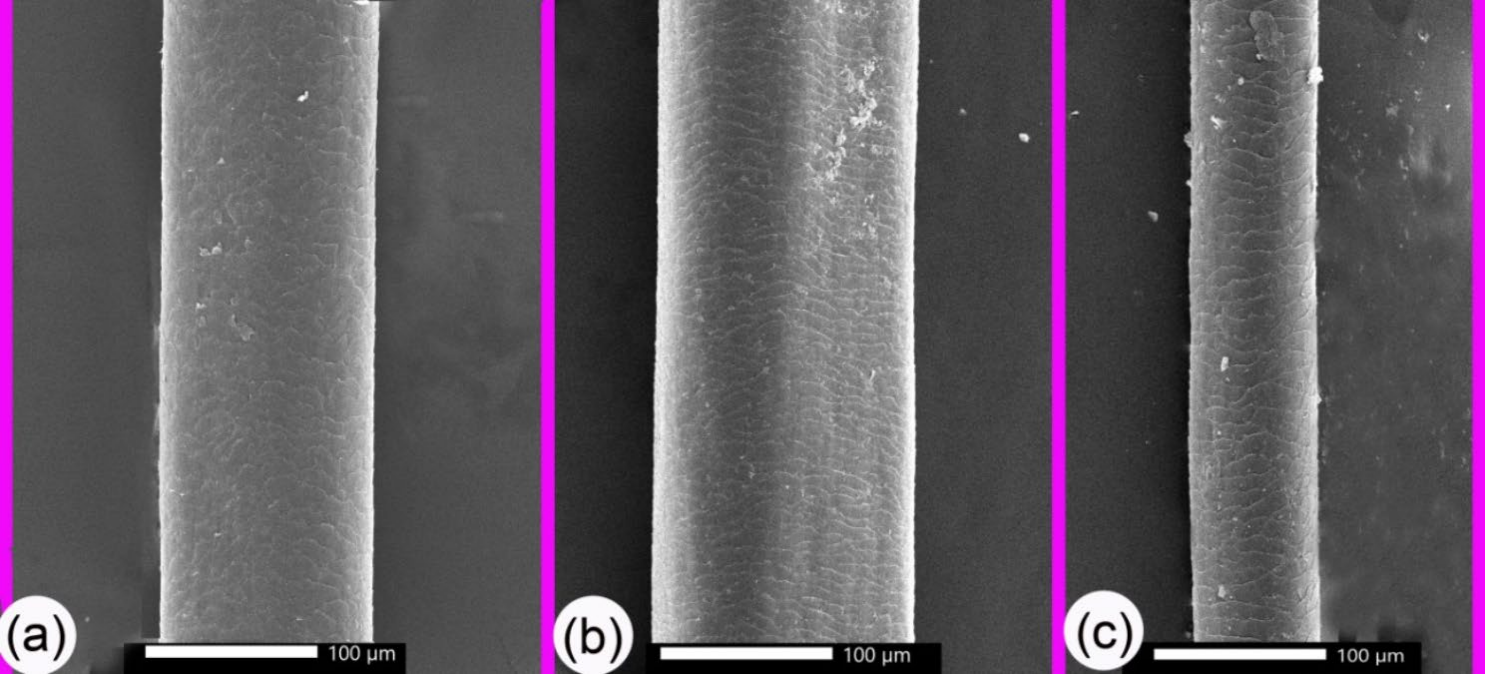
SEM of the hair shaft explained the width change with age. Panel (**a**) The hair shaft in the G1 camels had moderate widths. Panel (**b**) the hair shaft in the G2 camels had the largest widths. Panel (**c**) The hair shaft in the G3 camels had the smallest widths

**Fig. 6:**
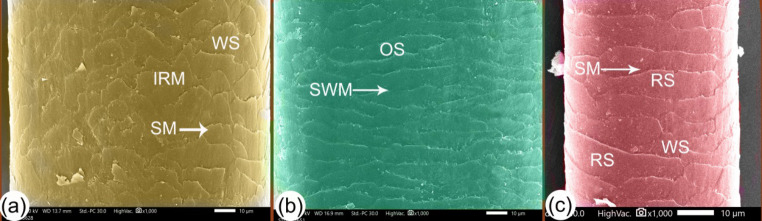
SEM explained the cuticle scales of the camel hair. Panel (**a**) shows G1 camel hair had wavy scales like mountain tips (MS) with some irregular margins (IRM) whilst others with smooth margins. Panel (**b**) shows G2 camel hairs had long oval shape scales (OS) with smooth wavy margins. Panel (**c**) shows G3 camel hairs had long rectangular shape scales (RS) with smooth margins (SM) and wedge shape scales (WS)

**Fig. 7:**
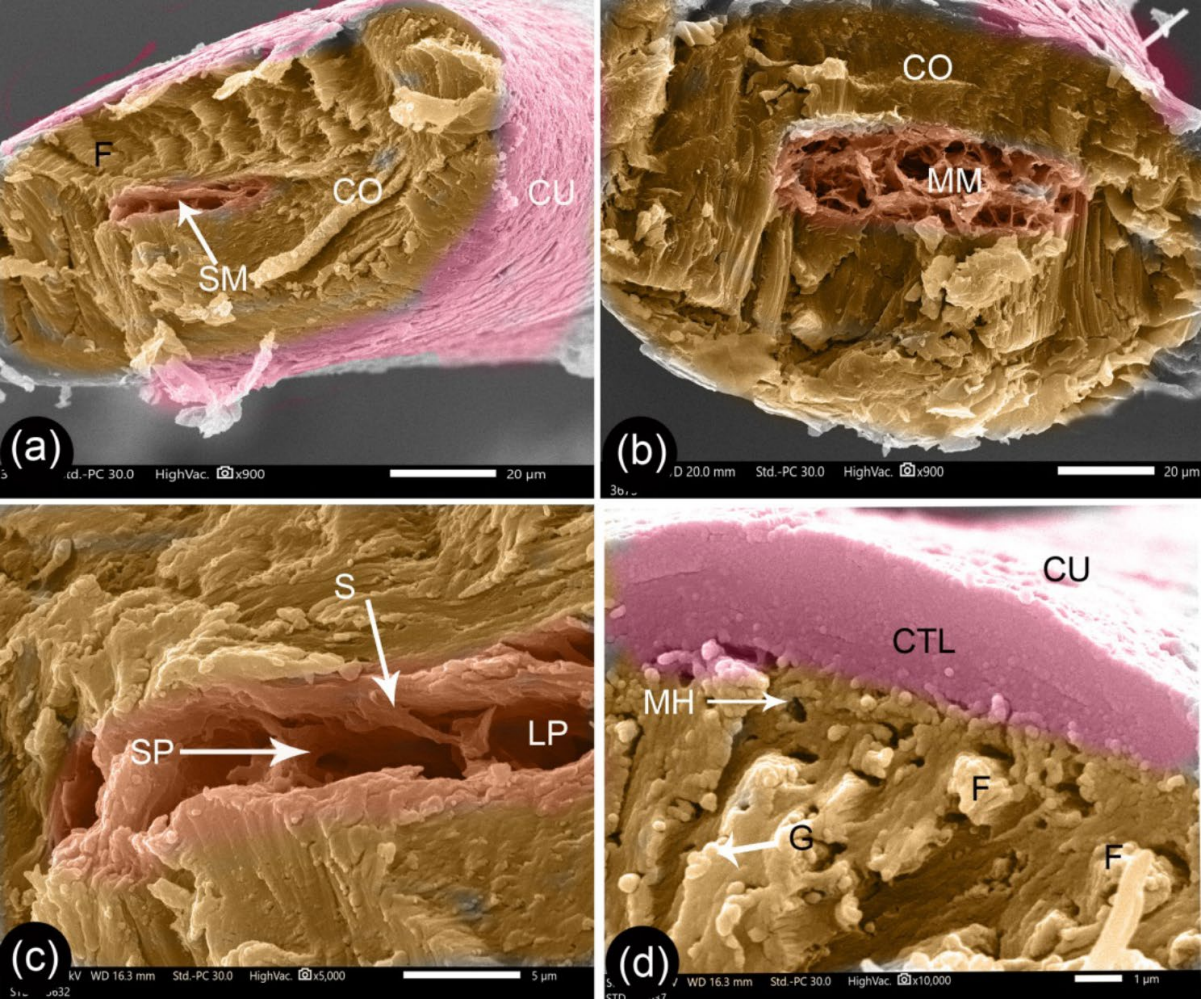
SEM of the cross-sections of G1 camel hair. Panels (**a** and **b**) exhibit the general structure of the hair. Panel (**c**) shows higher magnification of the vacuoles in the medulla. Panel (**d**) is a higher magnification of the cortex and wall of the cuticle. Hair cuticle (Cu), hair cortex (Co), small-sized medulla (sM), fibers of the cortex (F), septa (S), large pore (LP), small pore (SP), pigment granules (G), minute holes (MH), and cuticle layers (CUL)

**Fig. 8:**
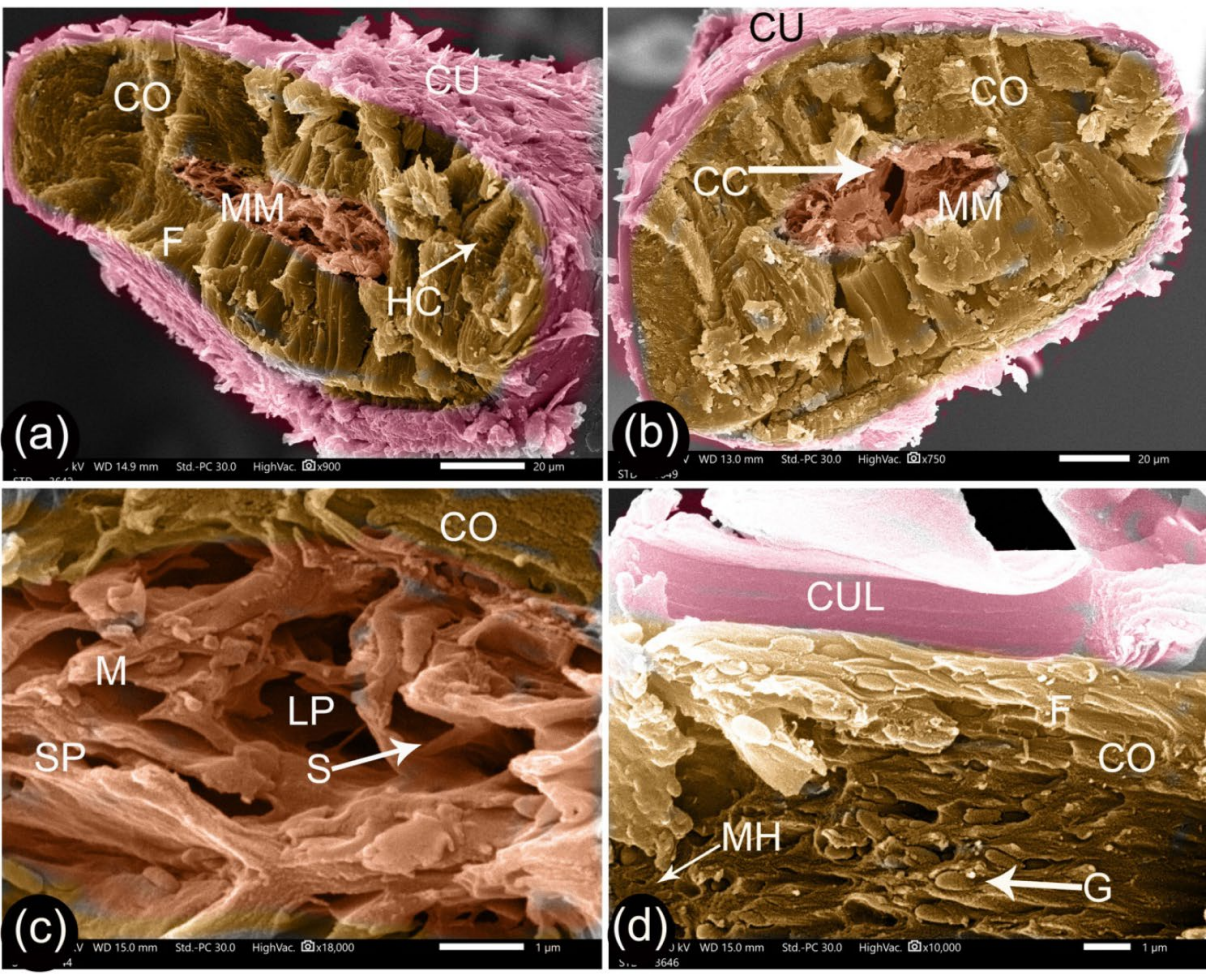
SEM of the cross-section of the G2 camel hair. Panels (**a** and **b**) exhibit the general structure of the hair. Panel (**c**) shows higher magnification of the vacuoles of the medulla. Panel (**d**) magnifies the cortex and wall of the cuticle. Hair cuticle (Cu), hair cortex (Co), medium-sized medulla (MM), hollow channels (HC), central canal (CC), fibers of cortex (F), vacuolated medulla (M), septa (S), large pore (LP), small pore (SP), pigment granules (G), minute holes (MH), and cuticle layers (CUL)

**Fig. 9:**
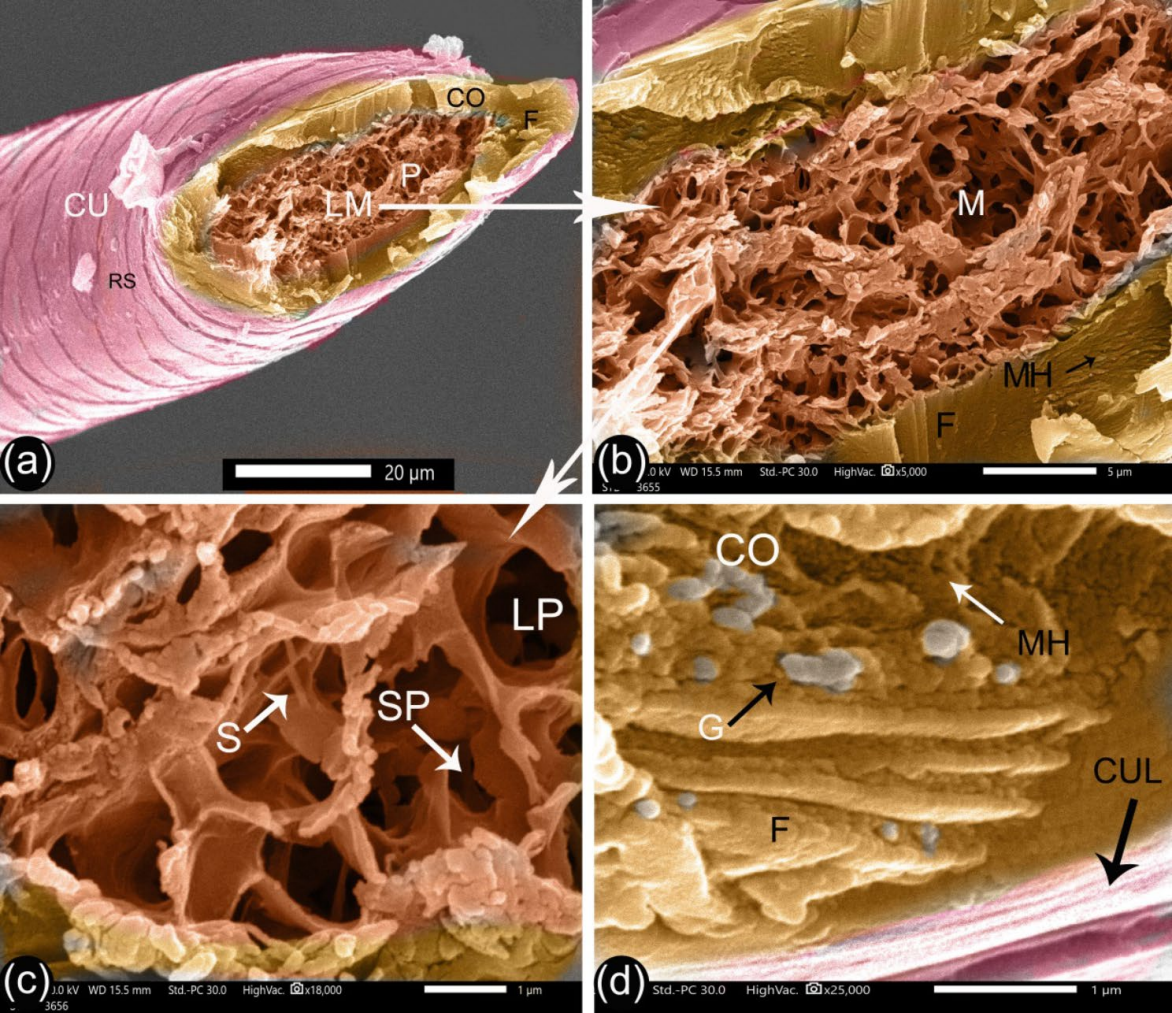
SEM of the cross-section of the G3 camel hair. Panels (**a** and **b**) show the general structure of the hair. Panel (**c**) magnifies the vacuoles of the medulla. Panel (**d**) magnifies the cortex and wall of the cuticle. Hair cuticle (Cu), hair cortex (Co), large-sized medulla (LM), long rectangular shape scale (RS), pores (P), fibers of the cortex (F), vacuolated medulla (M) septa (S), large pore (LP), small pore (SP), pigment granules (G) and cuticle layers (CUL)

The size of the hair and the shape of the cuticle also changed with age. The variation in width with age was measured via SEM examination of the hair shaft (Table 2 and Chart 2); the width was moderate at G1, maximum at G2, and smallest at G3 (Fig. 5a–c). The cuticle scales presented in a variety of shapes (wedged, oval, or rectangular; Fig. 5a–c). The scale positions were transverse, the pattern was imbricate (scales overlapped with each other) and consisted of a regular scale pattern, and the scale margins were irregular and occasionally smooth. G1 camels had wavy scale-like mountain tips with an irregular margin and another with a smooth margin (Fig. 6a). The hair scales of the G2 camels were mostly long oval-shaped scales with smooth wavy margins (Fig. 6b), whilst the scales in the G3 camels mostly had a long rectangular-shape with smooth margins, plus wedge-shaped scales (Fig. 6c). The wall cuticles also had many layers, ranging from 5 to 6 layers (Figs. 7, 8, and 9).

**Table 2:** Camel hair width of the shaft, medulla, cortex, cuticle, and scales measurements at the three different ages by SEM (mean ± standard error and p-value)

Measurements	G1	G2	G3	*p*-value
Diameter of the hair shaft (width) (µm)	108.74 ± 4.32^b^	150.88 ± 3.53^a^	56.95 ± 5.22^c^	0.0341
Highest scale (µm)	11.04 ± 0.30^a^	8.03 ± 0.95^b^	7.15 ± 0.46^c^	0.0401
Width of the scale (µm)	16.43 ± 1.29 ^b^	26.22 ± 3.3^a^	26.71 ± 2.34 ^a^	0.0332
Cuticle thickness (µm)	2.60 ± 0.13^a^	2.10 ± 0.15^b^	1.50 ± 0.14^c^	0.0153
Cortex thickness (µm)	59.81 ± 1.85^a^	33.67 ± 0.94^b^	4.02 ± 0.147^c^	0.0400
Medulla thickness (µm)	36.53 ± 2.02^c^	66.5 ± 2.3^a^	43.06 ± 3.4^b^	0.0357
Medulla index %	0.33 ± 0.02^c^	0.44 ± 0.03^b^	0.77 ± 0.05^a^	0.0432

Means within the same raw carrying different superscripts are significantly different at *P* < 0.05

The diameter of the cortex was opposite in terms of relationships to age to that observed for the diameter of the medulla, and it consisted of a longitudinal bundle of fibers. A minute-hole on the fiber surface and hollow channels appeared at the cross-section surfaces, which correspond to the fibrils that were pulled out (Figs. 7, 8, and 9). The medulla was a spongy-like structure with large pores divided by septa into small pores (Figs. 7, 8, and 9). The G1 camel medulla varied from a thin medulla with one or two rows of pores through to a moderate-sized medulla with many rows (Fig. 7a-b). G2 camels had a moderate-sized medulla with many rows of pores, a central small canal, and collateral rows of pores (Fig. 8a-b). Meanwhile, the medulla was larger in the G3 camels (Fig. 9a).

### SEM measurements

**Chart 3:**
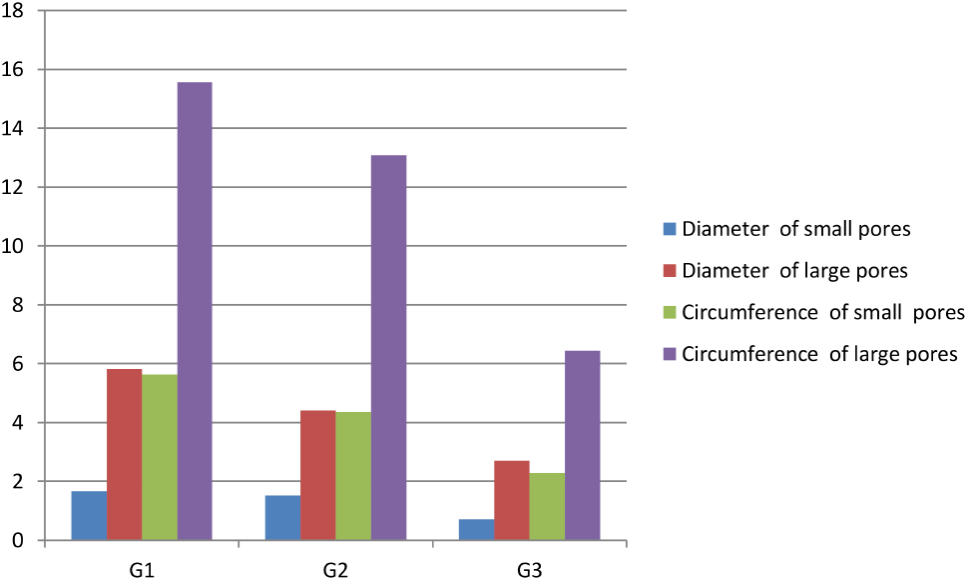
Changes in the camel hair medulla pore diameters and circumferences at three different ages analyzed using a light microscope. The G1 camel had the largest diameter and circumference of the small and large pores, while the G3 camel had the smallest diameters and circumferences of the small and large pores

Table 2 and Chart 2 show that the SEM measurements nearly matched the light microscopy measurement pattern but had lower values. The mean shaft diameter of the camel hair was still related to age; the highest diameter measurement (and significantly different) (p-value = 0.0341) was observed in G2 whereas G3 showed the lowest. The mean shaft diameters of the hairs were measured at 108.74 ± 4.32 μm in G1, 150.88 ± 3.53 μm in G2, and 56.972 ± 5.22 μm in G3 camels. The average distance between the successive scale values was significantly decreased from the youngest G1 through to the older G3 camels. It was measured at 11.04 ± 0.3 μm in G1 camels (with a significance difference) (p-value = 0401), 8.032 ± 0.95 μm in the G2 group, and 7.15 ± 0.46 μm in the G3 camels. At the same time, the width of the scale values were significantly increased (p-value = 0.0332) from the younger G1 through to the older G3 camels, measured at 16.43 ± 1.29 μm in G1 camels, 26.21 ± 3.3 μm in G2, and 26.7 ± 2.342 μm in G3 camels. The medullary index also had an apparent significant (p-value = 0.0432) relationship with age, similar to the results observed using light microscopy. The medulla width was at its greatest in G3 camels. Small and large pore diameters and circumferences had an inverse relation with age, as shown in Table 3 and Chart 3. The G1 camels had the largest diameters and circumferences of both the small (p-value = 0.0354 and 0.0501, respectively) and large pores (p-value = 0.0406 and 0.0411, respectively) with a marked significant difference. The G1 diameters of small and large pores respectively were 1.68 ± 0.146 μm and 5.82 ± 0.58 μm, and the circumferences were 5.64 ± 0.49 μm and 15.57 ± 0.69 μm, respectively. In contrast, in the older G3 camels, the diameters and circumferences of the small and large pores were 0.71 ± 0.086 μm and 2.7 ± 0.54 μm, whilst circumferences were 2.29 ± 0.048 μm and 6.44 ± 0.93 μm, respectively Table 4.

**Table 3:** Camel hair medulla pores diameter and circumference at the three ages by SEM (mean ± standard error and p-value)

Measurements	G1	G2	G3	*p*-value
Diameter of large pores	5.84 ± 0.58^a^	4.420 ± 0.50^a^	2.72 ± 0.71^b^	0.0406
Diameter of small pores	1.69 ± 0.19^a^	1.52 ± 0.18^a^	0.71 ± 0.08^b^	0.0354
Circumference of large pores	15.57 ± 0.58^a^	13.08 ± 1.16^b^	6.44 ± 0.893^c^	0.0411
Circumference of small pores	5.64 ± 0.49^a^	4.36 ± 0.39^ab^	2.29 ± 0.05^b^	0.0501

Means within the same raw carrying different superscripts are significantly different at *p* < 0.05

**Table 4:** Different elements of the camel hair cuticle and the medulla and cortex cross-section at the three different ages by SEM-EDX (mean ± standard error)

	Cuticle elements			Cross-section elements		
	G1	G2	G3	G1	G2	G3
C	45.36 ± 0.52	43.80 ± 0.31	43.67 ± 0.26	54.71 ± 0.26	47.09 ± 0.35	62.44 ± 0.36
O	34.56 ± 1.11	31.65 ± 0.63	32.33 ± 0.54	31.97 ± 0.56	31.36 ± 0.70	30.57 ± 0.60
N	16.92 ± 1.07	20.87 ± 0.64	20.47 ± 0.54	12.72 ± 0.56	17.82 ± 0.71	1.66 ± 0.45
S	2.62 ± 0.14	3.67 ± 0.09	3.52 ± 0.08	0.54 ± 0.04	2.84 ± 0.09	1.47 ± 0.06
Ca	0.54 ± 0.09	–	–	0.06 ± 0.02	–	1.14 ± 0.07
AL	–	–	–	–	0.90 ± 0.06	1.51 ± 0.07
Si	–	–	–	–	–	0.97 ± 0.06
K	–	–	–	–	–	0.25 ± 0.04

### SEM-EDX analysis

**Fig. 10:**
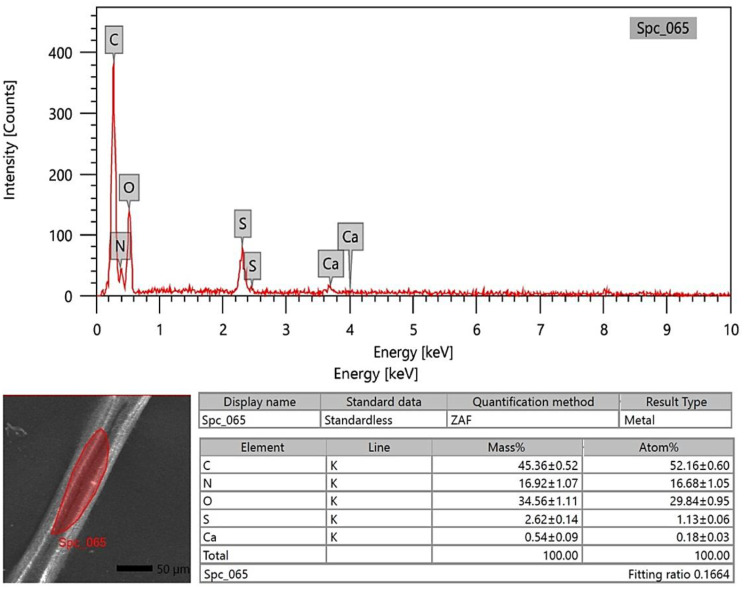
SEM-EDX image showing the examined area of the cuticle of the hair of the G1 camel. The hair cuticle without gold covering by SEM with examined area highlighted in red; the chart and table of elements show the mass of each element; the highest elements were carbon, oxygen, nitrogen, sulfur, and calcium, respectively

**Fig. 11:**
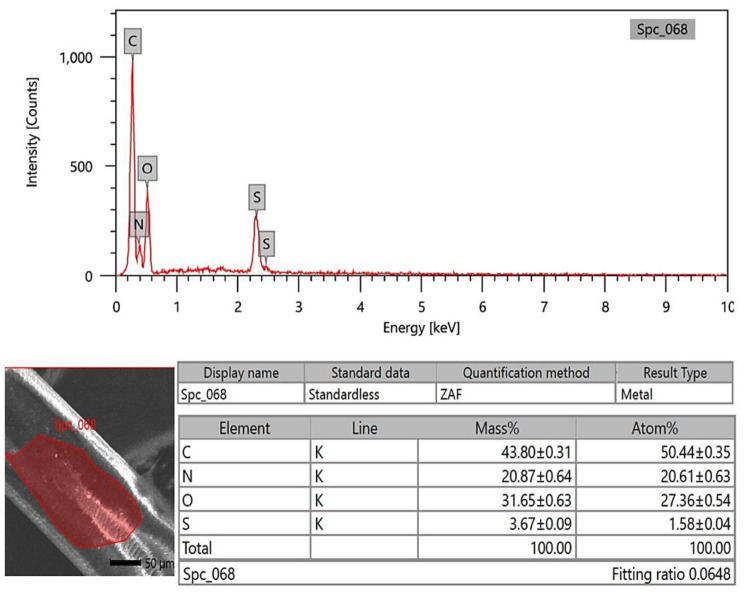
SEM-EDX image showing the examined area of the cuticle of the hair of the G2 camel. The hair cuticle without gold covering by SEM and the examined area is shown with a red highlight; the chart and table of elements show the mass of each element; the highest elements were carbon, oxygen, nitrogen, and sulfur, respectively

**Fig. 12:**
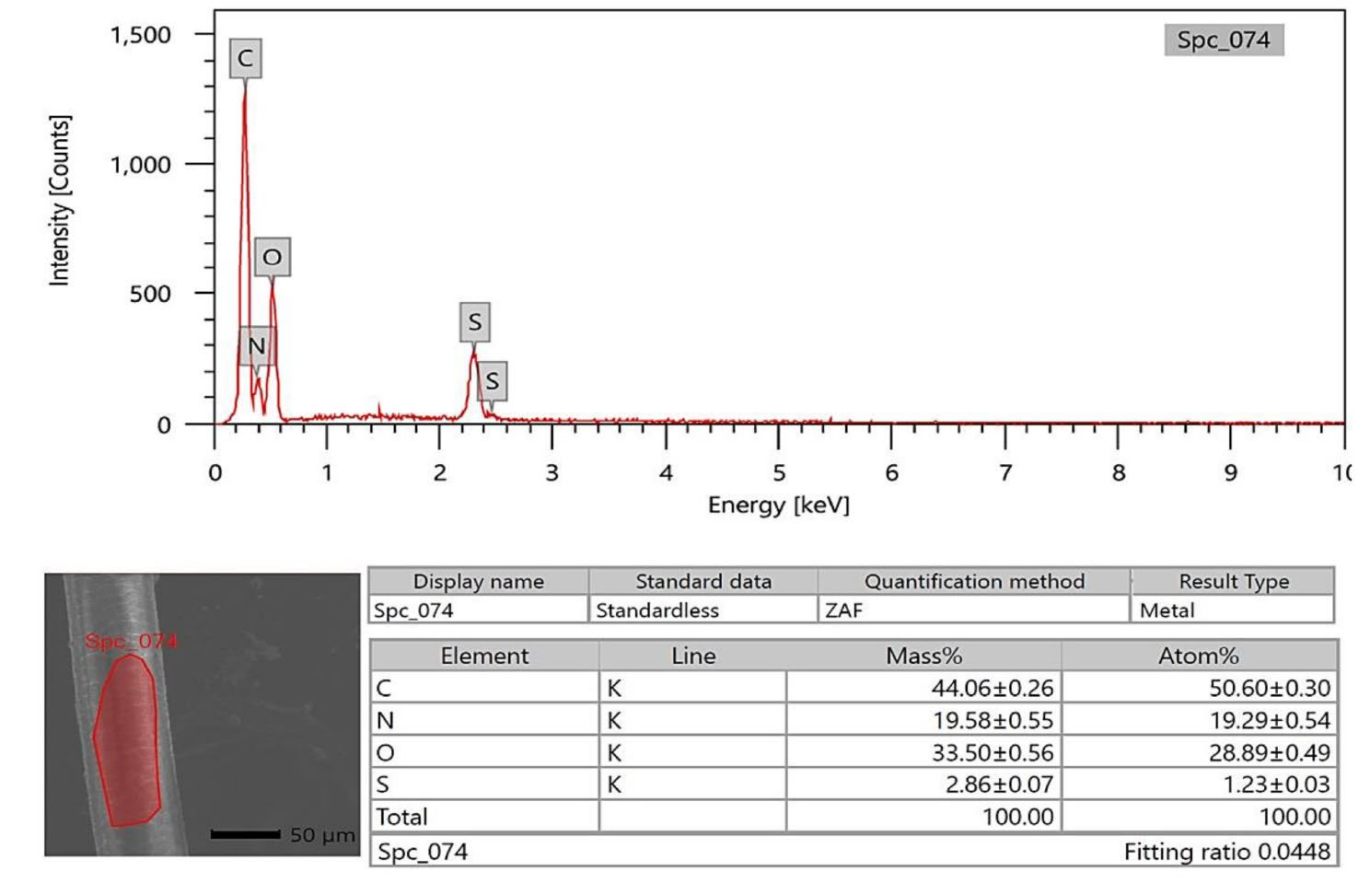
SEM-EDX image showing the examined area of the cuticle of the hair of the G3 camel. The hair cuticle without gold covering by SEM with the examined area highlighted in red; the chart and table of elements show the mass of each element; the highest elements were carbon, oxygen, nitrogen, and sulfur, respectively

**Fig. 13:**
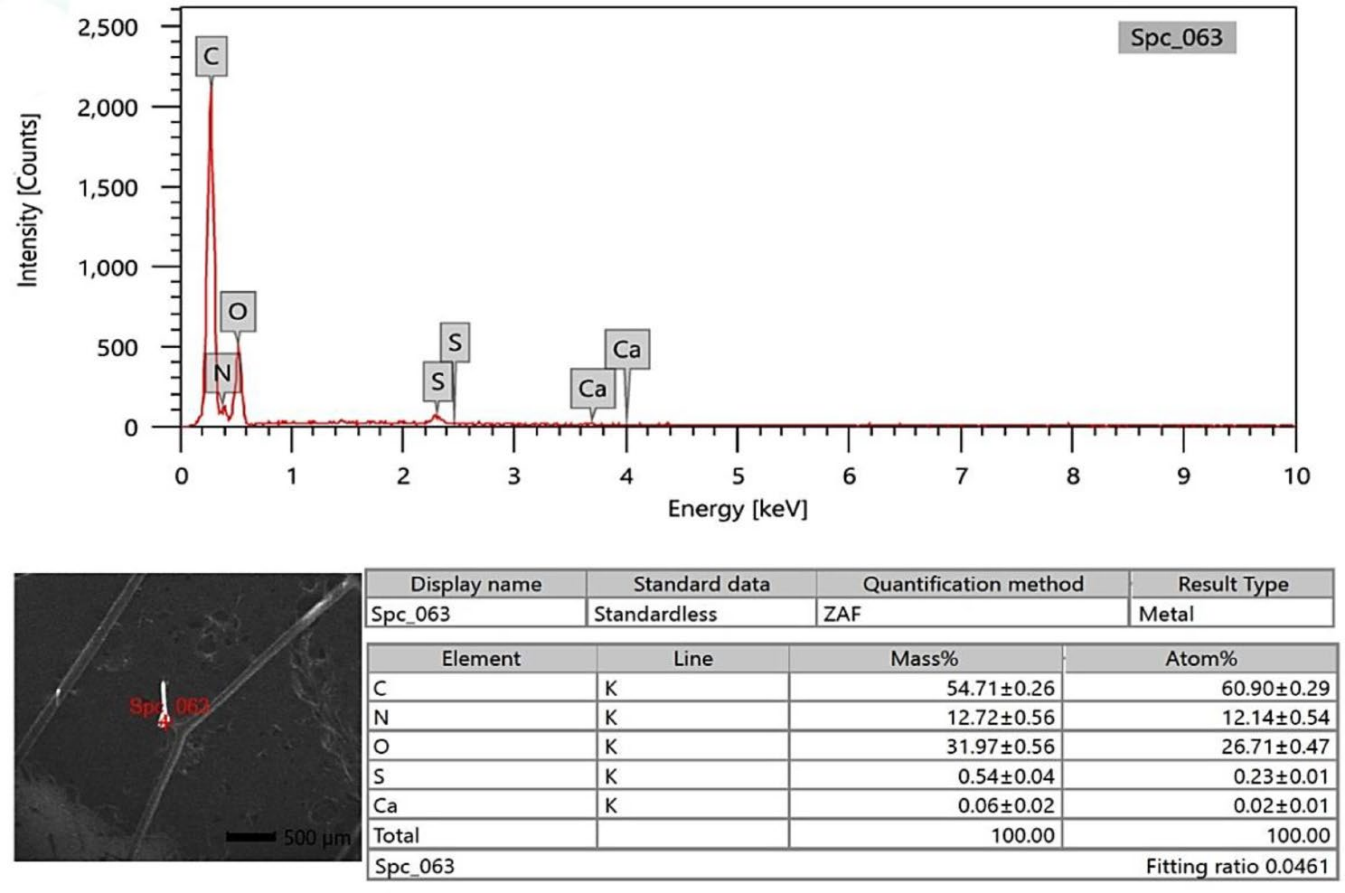
SEM-EDX image showing the examined cross-section area of the hair of the G1 camel. The figure showed the hair cross-section without gold covering by SEM with the examined area shown in red. The chart and table of elements showed the mass of each element. The highest elements were carbon, oxygen, nitrogen, sulfur, and calcium, respectively

**Fig. 14:**
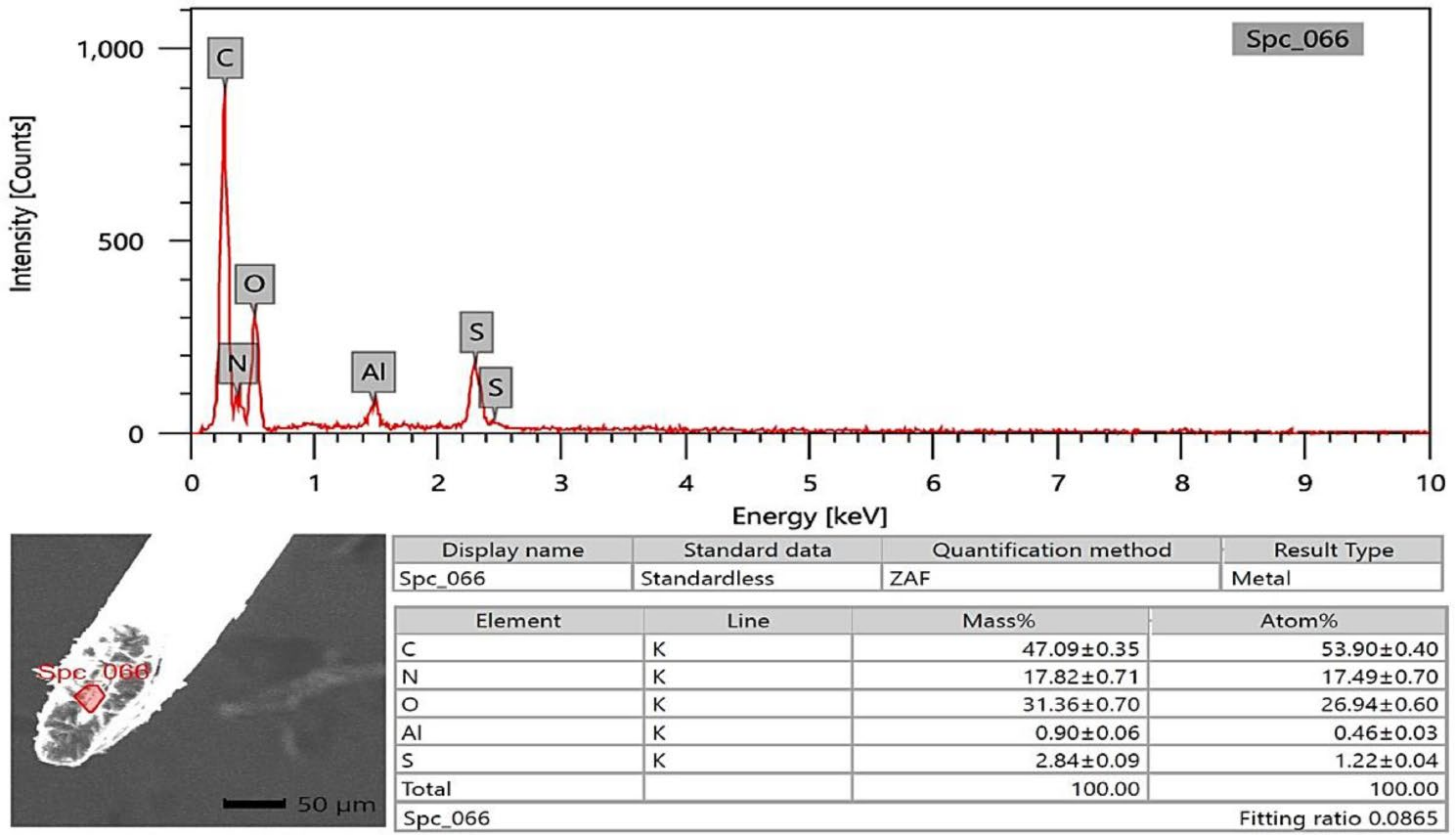
SEM-EDX image showing the examined cross-section area of the hair of the G2 camel. The figure showed the hair cross-section without gold covering by SEM with the examined area indicated in red. The chart and table of elements show the mass of each element. The highest elements were carbon, oxygen, nitrogen, sulfur, and aluminum, respectively

**Fig. 15:**
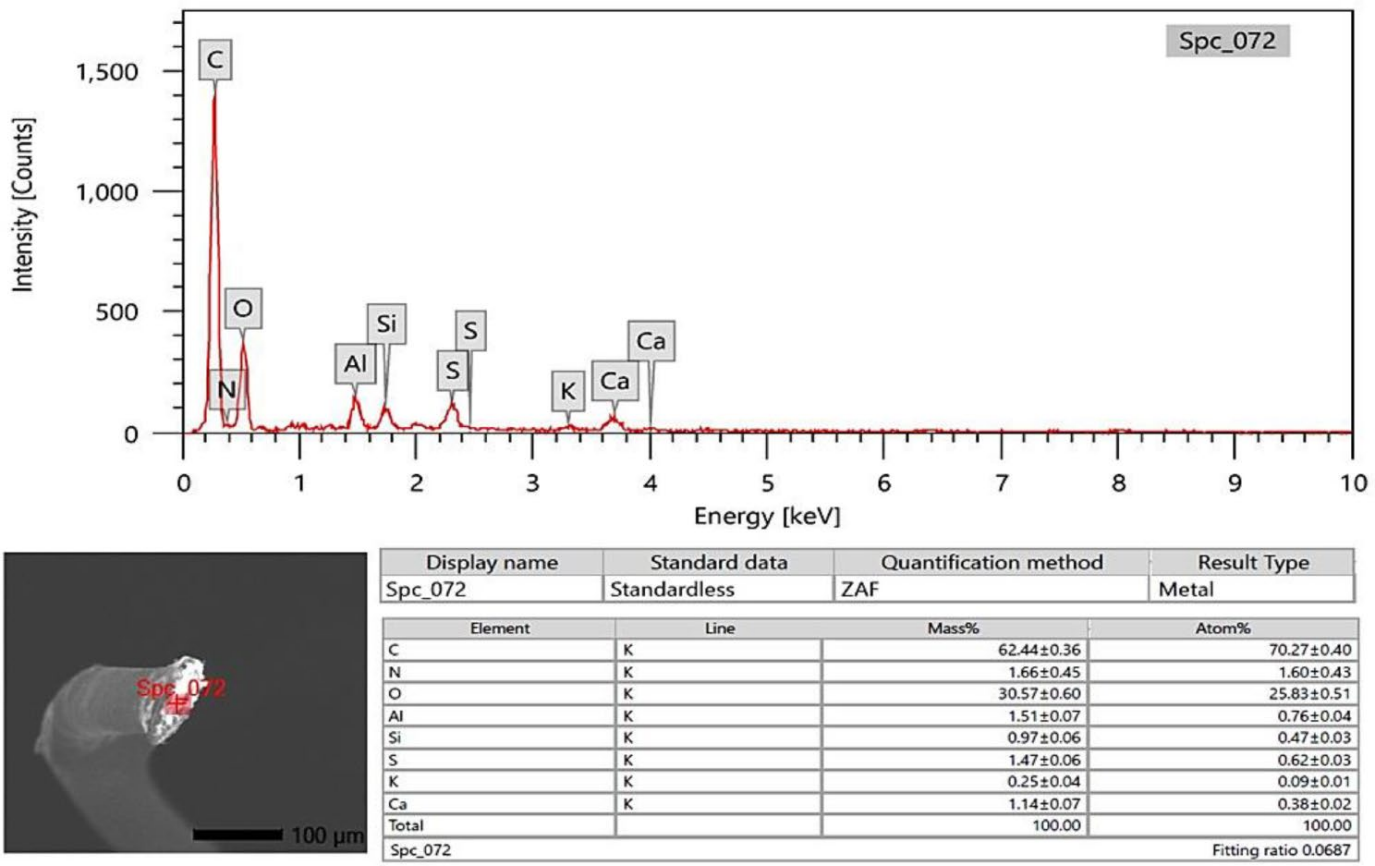
SEM-EDX image showing the examined cross-section area of the hair of G3 camel. The hair cross-section without gold covering by SEM with the examined area indicated in red. The chart and table of elements show the mass of each element; the highest elements were the carbon, oxygen, nitrogen, sulfur, aluminum, calcium, silicon, and potassium respectively

The photon energy dispersive X-ray spectra of four specimens of varying ages subjected to a 20 kV accelerating voltage yielded a micro-elemental picture of the hair cuticle and internal structure. The elements were identified in the EDX spectra by mass and atom percentages. EDX spectra recognized and detected the following via mass percentages: Carbon, oxygen, nitrogen, and sulfur, as well as calcium, were identified at the hair cuticle in the G1 camel (Figs. 10, 11 and 12), and carbon, oxygen, nitrogen, sulfur, calcium, aluminum, silicon, and potassium were present in the cross-sectional element (medulla and cortex) (Figs. 13, 14 and 15). In the examined samples, the cuticle carbon mass decreased very slightly with increasing age: 45.36 ± 0.52%, 43.80 ± 0.31%, and 43.67 ± 0.26%, and oxygen was mainly variable, with sulfur having the highest value of 3.67 ± 0.09 at 3–5 years but the lowest value in G1. There was a significant increase in carbon percentages (62.44 ± 0.36%) and a significant decrease in nitrogen percentages (1.66 ± 0.45%) at G3, which could be attributed to the cortex and spongy shape of the medulla occupying portions of the cross-section, and the percentage of sulfur present also increased in the G2 age range. Carbon and nitrogen percentages were significantly higher in the cross-section than in the cuticle. In comparison, the rates of oxygen and sulfur in the cuticle were markedly higher than the readings in the cross-sections of hair. At the three different ages, there were correlations between the medullary index and the percentages of carbon and nitrogen (Figs. 13, 14 and 15).

## Discussion

In this study, the color of the hair on the hump varied from light to dark brown, and the medulla pattern and structures of the hair in the camels were varied but mainly continuous and amorphous. The light and SEM measurements indicated a greater correlation between the medulla index, the cortex, and cuticle widths with increasing age. The shapes of the cuticle scales varied between the three groups. Finally, the SEM-EDX spectra recognized the elements in the hair cuticle, cortex, and medulla.

In this study, the hair on the camel hump ranged from light to dark brown. The hair quality of the Maghrebi one-humped camel was previously reported to be rough and thick, and in the Hamra Maghrebi camel it was a brown color [38]. Color is a key feature used to classify camels, and three major breeds of Saudi camels have been distinguished by their coat color: Magaheem, Magateer, and Al-Homr or Al-Sofr [11]. Accordingly, it has been discovered [39] that the Arabian Peninsula has twelve camel phenotypes and breeds. The Mezayen camel breed had a color class (main color) in which many varieties existed. There are six color classes: black (Majaheem), smoky-brown (Sofor), brown (Shaele), red (Homor), wheat (Shageh), and white (Waddah) [40, 41]. Several subtypes within each breed correspond to subtle differences in coat color and tone [42, 43].

Generally, every domestic animal has a characteristic hair pattern [44]. In our study, the one-humped camel hair medulla had a variety of patterns and structures, including a continuous pattern and an amorphous structure (75%, 70%, and 90% in the G1, G2, and G3 groups, respectively), as well as interrupted intruding amorphous, interrupted, vacuolated, or amorphous medulla and fragmented amorphous medulla (25%, 30%, and 10% in the G1, G2, and G3 groups, respectively). Previous research has shown that the medulla of Maghrebi camel hair fibers was 50% continuous, 38% fragmented, and 12% interrupted [45]. The adult male one-humped camel’s cortex was pigmented, and the medulla had a continuous, smooth margin [13] and camel hair is divided into two parts: thin, non-medullated, or medullated hair fibers and thick, interrupted, or continuous medulla hair fibers [24]. Comparatively, the dog’s medulla is an exception and is comprised multicellular cornified cells [46], the medullary structure extended into the root and contained traces of follicular tissue. Suiformes have thick-walled medullas that do not form columns, whereas Bovidae and Carnivora have variable-mesh medullas [47]. The thickness and thickness of the medulla’s ladder and non-columnar morphologies distinguish them [48].

In the present study, hair measurements were taken using light microscopy and SEM to compare camels in different age groups. The medulla index in the three camel groups increased with age, from 0.298 to 0.77. The main ratio was also determined by age. The shaft width increased from G1 to G2 but decreased significantly for the older G3 camels. The cortex and cuticle widths narrowed with age.

By light microscopy, the thickness of the hair fibers varies depending on sex and camel age [49]. The morphology of the hair fibers in the present study revealed distinct shapes in the cortex and medulla. The fibers of the dehaired dromedary hairs had a relatively low average diameter in young camels, which increased with age; thus, young camel hair can be used for handicrafts, while adult camel fleece can be used to make tents for example. The medullary index in camels is also varied; in Bactrian camels, the medullary index was less than 1/4 of the hair diameter (16.63 to 16.86 μm), whereas, in the Llama, the medullary index was about 1/3 of the hair diameter, ranging from 33.33 μm to 33.96 μm [50]. Camel hair had a medullary index percentage of approximately 78.66% [45]. The medulla within adult male dromedary camels was found to be more than half the diameter of their hair [13]. The shaft width increased in our study from G1 to G2, from 159.4 ± 7.529 μm to 166.48 ± 20.79 μm, whilst the shaft width decreased in the older G3 camels to about one-third 60.416 ± 6.730 μm of that seen in the younger ages. In camel breeds residing in Sudan, the hair shaft width ranged from 29.92 to 70.40 μm at one-year-old camel and 139 to 142.6 μm in the six-year-old camel [49]. SEM analysis has revealed that Bactrian camel hairs ranged in diameter from 42.66 to 44.23 μm [50], whilst alpaca hair has an average diameter of 32.27 ± 4.06 μm [51]. However, hair shaft width in buffalo, horse, goat, dog, and cat ranged from 23.78 ± 1.24 μm to 85.51 ± 1.14 μm [52]. In the present study, the cortex and cuticle width decreased with age. The cortex in buffalo increased with age, while the cuticle decreased slightly [16].

In this study, the high diameter of the hair in the G1 to G2 camels makes their wool more suitable for carpets and handicrafts, whereas the older G3 animals produce a smaller diameter of hair that may be used for outerwear. The dromedaries’ habitats vary in terms of climate, from the dry, semi-arid, or desert areas of Africa. Camel’s wool is a valuable and a required product, and its value rests mainly on the thickness and length. The most valuable are thinner fibers which make a soft and attractive material for scarves and sweaters of the highest quality. Fibers of medium thickness may be used to produce outerwear clothing. Thick wool finds its way into manufacturing tents, carpets, blankets, ropes, halters, and insulating material [53]. The Tunisian camel wool is often used to produce handicrafts and carpets [54].

In our study, the cuticle scales overlapped and were varied in shape; its appearance was similar to wavy like mountains in one-year-old camels, had long oval shapes with smooth wavy margins in G2 camels, and long rectangular or wedge shapes were common in G3 camels. It was previously discovered that camel hair fiber scales are mosaic in wool [45]. The camel’s scale pattern was imbricate, the margin was crenate, and the scale had irregular waves with a moderate distance [13, 48]. Camel hair scales have been reported as having an irregular mosaic pattern and are smooth [49]. In ruminants, the scaling pattern was imbricated with no protrusions from the hair shaft, whereas in dogs, the scales were not prominent [23].

SEM-EDX elements found in the hair cuticle included carbon, oxygen, nitrogen, sulfur, and calcium. In contrast, the elements found in the medulla and cortex cross-section were carbon, oxygen, nitrogen, sulfur, calcium, aluminum, silicon, and potassium. Carbon mass decreased slightly with age in the cuticle, in line with a previous report in the buffalo [16]. In buffalo, the following elements were identified: calcium, potassium, iron, magnesium, phosphorus, silicon, sulfur, and zinc [52]. EDX spectra revealed that carbon, oxygen, and nitrogen were more abundant in all age groups of buffalo. In buffalo hair, oxygen was the second most abundant element after carbon. The carbon mass in the examined samples decreased slightly with age (42.31%, 39.18%, 38.88%, and 38.49%) [16]. Oxygen was the second highest element in the goat hair cuticle [36], and the following elements were also found in the hair cuticle (C, O, S, Na, Ca, K, Co, Cu, Fe, Mn, Mg, and Zn). The elemental sodium, potassium, sulfur, and calcium levels were determined using SEM-EDX on three *Felidae* animals [17]. EDX has also already been used with SEM to investigate these structures’ elemental concentration and distribution profile in people [55]. The increase in carbon mass with aging in the camels in the present study may be due to the loss of inorganic material from the hair. The accuracy of hair mineral analysis remains controversial [56]. Some hair elements are specific for each animal, such as bromine in sheep and magnesium and phosphorus in buffalo. In addition, vanadium and titanium were found only in cattle, dogs, and sheep, and the lowest calcium was found in cattle [52]. The mineral content of wool in sheep depends on the physiological status, such as parturition, mating, and gestation [57]. It has been demonstrated that breed differences exist in some elements of hair mineral content, even when nutrition is similar [58].

Finally, due to the study’s limitations, in the future we would like to create more groups and examine more hair samples and include different parts of the hair, such as the apex and base, naturally the expense of SEM and EDX analysis means more funds are required.

## Conclusion

Using light microscopy, SEM, and SEM-EDX, we found that dromedary camel hair had a pigmented cortex and varied patterns and structures of the medulla from the one-year-old compared to the ten-year-old camels. Pigment granules of various sizes were present on the hair cortex. Small and large pores were identified on the hair medulla. The medulla displayed continuous, interrupted, and fragmented patterns at different ages, and vacuolated, intruding, and amorphous structures, and regular or irregular margins were observed. The cuticles’ scales, shapes and diameters varied across the different ages. The cortex’s diameter was opposite to the diameter of the medulla – a smaller cortex was observed alongside a larger medulla and vice versa. The medulla index increased with age, while the cortex and cuticle width decreased. SEM-EDX spectra recognized and detected carbon, oxygen, and nitrogen, which were more abundant in all groups. Hair measurements varied according to age, as did scale microstructure, and elemental composition. The Maghrebi dromedary camel’s three age stages correlated to differing hair structure and the percentage of carbon and nitrogen.

## Data Availability

The datasets used and analyzed during the current study are available from the corresponding author upon reasonable request.

## Abbreviations

G1: one-year-old camels
G2: 3–5-year-old camels
G3: 8–10-year-old camels
LM: Light microscopy
SEM: Scanning electron microscopy
CPD: Critical Point Drying
SEM-EDX: Scanning electron microscopy-Energy Dispersive X-Ray Analysis
ZAF: Correction means a correction that considers the following three effects on the characteristic X-ray intensity when performing quantitative analysis: (1) atomic number effect (Z), (2) absorption effect (A), and (3) fluorescence excitation effect (F).
C: Carbon
O: Oxygen
N: Nitrogen
N: Nitrogen
S: Sulfur
Na: Sodium
Ca: Calcium
P: Phosphorus
K: Potassium
Co: Cobalt
Cu: Copper
Fe: Iron
Al: Aluminum
Si: Silicon
Mn: Manganese
Mg: Magnesium
Zn: Zinc
